# Involvement of the left insula in the ecological validity of the human voice

**DOI:** 10.1038/srep08799

**Published:** 2015-03-05

**Authors:** Yuri Tamura, Shinji Kuriki, Tamami Nakano

**Affiliations:** 1 Dynamic Brain Network Laboratory, Graduate School of Frontiers Biosciences, Osaka University, Osaka, Japan; 2 Center for Information and Neural Networks (CiNet), National Institute of Information and Communications Technology and Osaka University, Osaka, Japan

## Abstract

A subtle difference between a real human and an artificial object that resembles a human evokes an impression of a large qualitative difference between them. This suggests the existence of a neural mechanism that processes the sense of humanness. To examine the presence of such a mechanism, we compared the behavioral and brain responses of participants who listened to human and artificial singing voices created from vocal fragments of a real human voice. The behavioral experiment showed that the song sung by human voices more often elicited positive feelings and feelings of humanness than the same song sung by artificial voices, although the lyrics, melody, and rhythm were identical. Functional magnetic resonance imaging revealed significantly higher activation in the left posterior insula in response to human voices than in response to artificial voices. Insular activation was not merely evoked by differences in acoustic features between the voices. Therefore, these results suggest that the left insula participates in the neural processing of the ecological quality of the human voice.

The landmark science fiction movie “Blade Runner” presents androids whose appearances are indistinguishable from those of real humans. However, with the present state of technology, it is difficult to create such humanoid robots. It is ironic that when a robot appears similar to a real person, a subtle difference can cause feelings of repulsion, which is a phenomenon known as the “uncanny valley.”^1^,^2^ suggesting that people have a unique sense of humanness.

Thus far, to the best of our knowledge, this phenomenon has only been investigated by measuring behavioral and neural responses to humanoid robots whose appearances resembled those of a human^3^,^4^. However, in terms of cost and technical complexity, it is very difficult to create humanoid robots whose appearances and movements resemble those of humans. Thus, it is difficult to examine the sense of humanness without eliciting a feeling of awkwardness in the person who perceives a humanoid robot. In contrast, the technology for synthesizing an artificial voice has advanced to a point that it can be used in our daily life as a tool for voice-guided navigation. Moreover, songs sung by an artificial voice are currently popular in Japan and have reached the top of the music charts. Therefore, we investigated the neural correlates of the sense of humanness by solely focusing on sound. We used an artificial voice created by singing-voice synthesizer technology, which uses a database of vocal fragments sampled from a single person. Because the singing voice contains an actual human voice, the physical characteristics between human and artificial voices are quite similar. Moreover, it is possible to make songs identical for human and artificial voices with regard to lyrics, melody, and rhythm. In comparison with using a speaking voice, it is extremely advantageous to use a singing voice for producing an identical rhythmic structure of language. Thus, we inferred that the difference in impressions between human and artificial voices may be derived from differences in the sense of humanness of each voice.

In this study, participants listened to 15-s segments of 12 Japanese songs, each sung two times: once by human voices and once by artificial voices (24 different stimuli in total). We first compared the participant impressions of human-likeness, positive feelings, and musical characteristics for the same songs sung by human and artificial voices; these factors were rated with a 10-item questionnaire. Using functional magnetic resonance imaging (fMRI), we also investigated the neural mechanisms related to the perception of humanness by comparing brain activation patterns elicited by human and artificial voices.

## Results

### Sound Analysis

**Figure 1:**
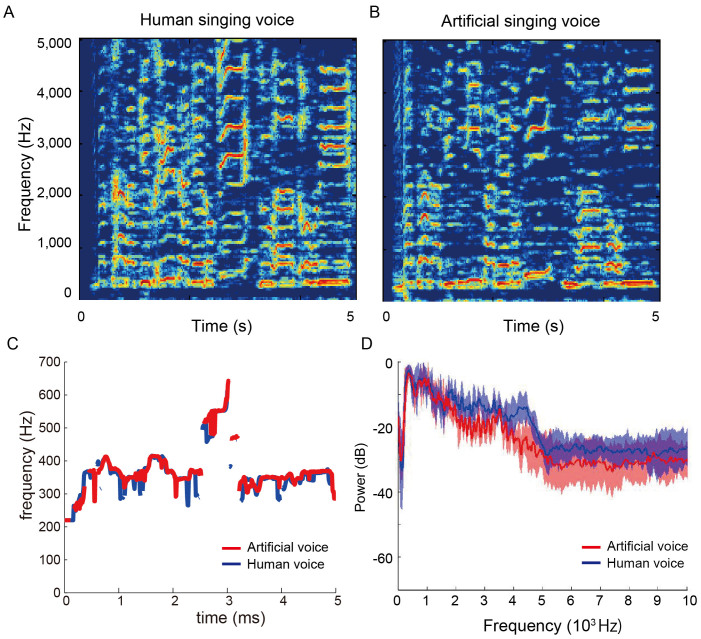
Temporal profiles of the music used in the experiment. (A–B) Spectrograms of the same song sung by human (A) and artificial (B) voices. (C) Fundamental frequencies of the same song sung by human (blue line) and artificial (red line) voices. (D) Average long-term average spectrum (LTAS) over all songs for the human (blue line) and artificial (red line) voices. The blue and red areas represent the range of a standard deviation.

We first compared the acoustic features between human and artificial voices and found that for the same melody, human and artificial voices revealed similar temporal profiles for the fundamental frequencies in each song (Figure 1A–C). In contrast, human voices had a lower sound level for the long-term average spectrum (LTAS) in the low frequency range (under 1 kHz) than the artificial voices; human voices also had a higher sound level in the high frequency range (2–5 kHz; Figure 1D and Table 1). A previous study demonstrated that the LTAS sound level in the high frequency range affected the quality of voice speech^5^. However, the alpha ratio was not markedly different between human and artificial voices because the variance among songs was larger than the difference between human and artificial voices.

### Behavioral Experiment

**Figure 2:**
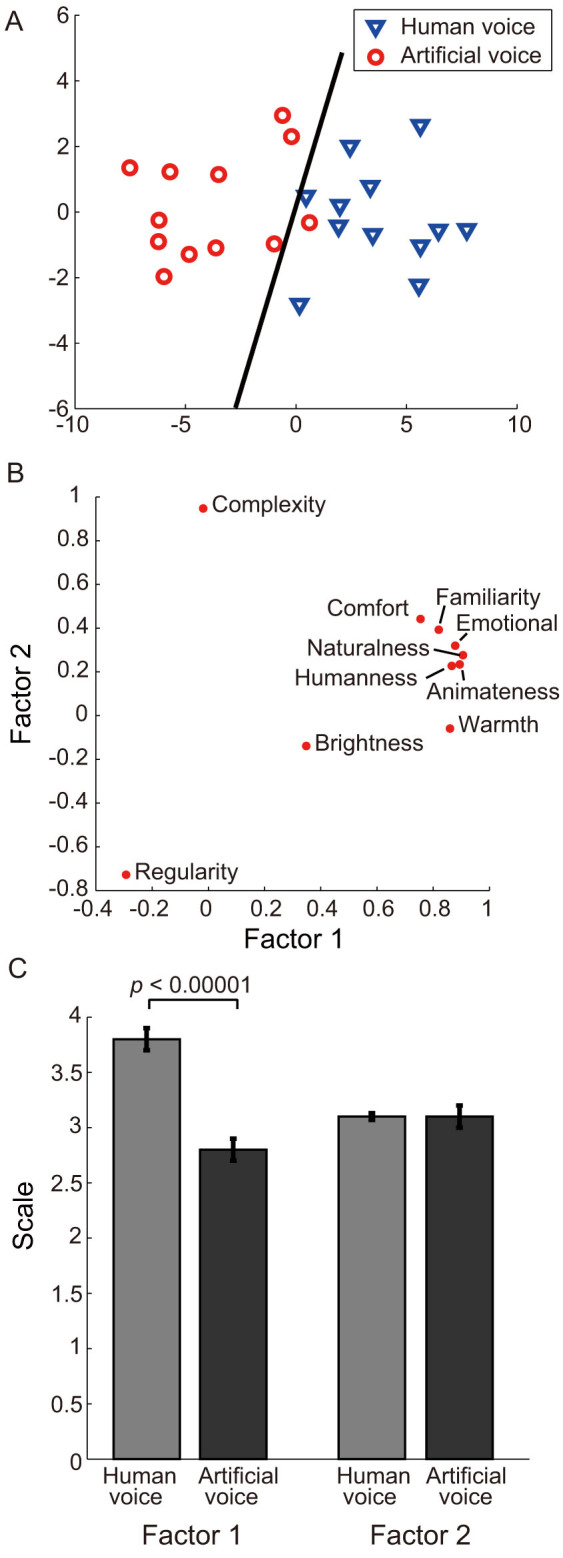
Behavioral results from the factor and cluster analysis. (A) Distributions of the response patterns of each song in the multidimensional scale plane. Each symbol represents a song sung by human (blue triangle) or artificial (red circle) voices. The black line discriminates human and artificial voices and was calculated using a linear discriminant analysis. (B) A graphical representation of the two factors analyzed with the ten questionnaires using factor analysis. (C) Comparison of the mean rating for each factor between human and artificial voices. The error bar represents standard error.

First, we conducted a cluster analysis using a multidimensional scale (MDS) to verify if the songs sung by human voices could be distinguished from those sung by artificial voices based on the response to each questionnaire item. As shown in Figure 2A, the songs sung by human voices (blue triangles) were distributed in the right plane and those sung by artificial voices (red circles) were distributed in the left plane. Linear discriminant analysis revealed that the two groups could be distinguished with an accuracy of more than 95% (black line).

Next, the participant impressions of the songs sung by a human or artificial voice were compared for each questionnaire item. A two-way analysis of variance identified a significant association between the voice (human vs. artificial) and questionnaire (F_9, 171_ = 43.1, *p* < 0.0001). As shown in Table 2, post hoc paired *t*-tests indicated that the mean ratings for the human voice were significantly higher than those for the artificial voice for items related to the impression of human-likeness (humanness, animateness, and naturalness) and the positive feelings elicited by the songs (emotion, familiarity, warmth, and comfort) (*p* < 0.0001, Bonferroni corrected). In contrast, there were no marked differences between the mean ratings of human and artificial voices for items related to the impression of musical characteristics (complexity, regularity, and brightness). We then extracted the factor structure underlying the questionnaire items using factor analysis. Two factors were identified from the 10 questionnaire items. As shown in Figure 2B and Table 2, impressions of humanness and positive feelings were combined into a single factor (Factor 1). In contrast, impressions of the musical characteristics comprised another factor (Factor 2). As shown in Figure 2C, the mean ratings for Factor 1 for human voices were significantly higher than those for artificial voices. All values are provided as mean ± standard deviation (SD) [human voice: 3.8 ± 0.1; artificial voice: 2.8 ± 0.1; t_13_ = 7.1, *p* < 0.000001]. In contrast, the mean ratings for Factor 2 did not markedly differ between the two types of voices (human voice: 3.1 ± 0.03; artificial voice: 3.1 ± 0.1).

### FMRI experiment

**Figure 3:**
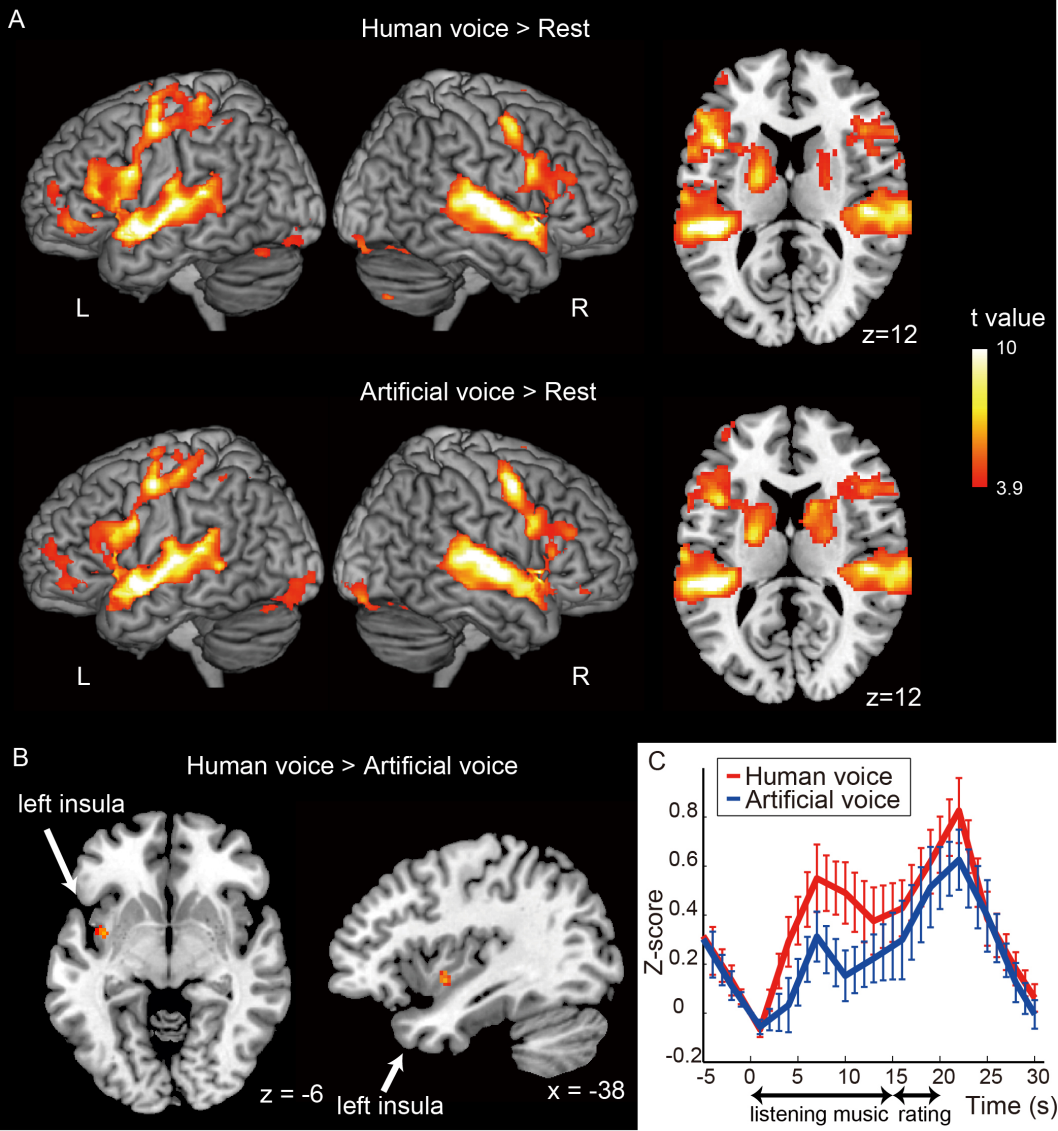
Comparison of activation between human and artificial voices. (A) Brain activation elicited by human (above) and artificial (below) voices was compared with the activity in the resting state. (B) Areas of greater activation with the human voice compared with the artificial voice, masked by human voice activation (p < 0.05). The left insula [Montreal Neurological Institute (MNI): x = −38, y = 0, z = −6) revealed markedly greater activation for the human voice. (C) Mean time course of the activation in the left insula across 13 participants. The human voice (red line) induced a greater increase in blood oxygenation level-dependent signals than did the artificial voice (blue line) while listening to music. The threshold of significance was set to p < 0.001, and the extent of the cluster size (k) was set to >20. Error bars represent one standard error of the mean.

Subsequently, we compared the brain activation patterns elicited between human and artificial voices. Both voices induced bilateral activation in the superior temporal gyrus (STG), inferior frontal gyrus, cerebellum, supplementary motor area, precentral gyrus, insula, and putamen (Figure 3A, Table 3). Further examination revealed that the left posterior insula [Montreal Neurological Institute (MNI): x = −38, y = 0, z = −6] had markedly higher activation in response to the human voice than in response to the artificial voice (Figure 3B and Table 3). No other region revealed marked differences in activation between the responses to artificial and human voices.

The blood oxygenation level-dependent (BOLD) signal responses in the left insula also showed higher activation while the participants were listening to the human voice than while listening to the artificial voice (Figure 3C). In contrast, the excluded participants who did not perceive any difference in humanness between the two stimuli failed to show increased activation in their posterior insula in response to the human voice (Supplementary Fig. S1).

Next, we examined whether the activation in the left posterior insula was elicited by differences in acoustic feature processing. The precuneus was considerably activated by the LTAS sound level in the 0–1-kHz range (t = 7.7; MNI: x = −14, y = −58, z = 32). In contrast, the anterior cingulate cortex was significantly activated by the LTAS sound level in the 3–4-kHz range (t = 4.5; MNI: x = −8, y = 26, z = 26). However, the insula did not show marked activation in response to acoustic feature measurements.

## Discussion

In the present study, we demonstrated that the human voice elicited positive feelings and feelings of humanness more often in participants than did the artificial voice, although the lyrics, melody, and rhythm were identical. This finding demonstrates that we have a sense of humanness with voice and physical appearance^1^,^2^,^6^. Moreover, this sense of humanness with voice is spontaneously accompanied by positive emotions.

The fMRI study indicated that both human and artificial voices activated the bilateral primary auditory, frontal, and temporoparietal auditory association areas. This was consistent with the findings of previous brain imaging studies that examined brain activation patterns in participants listening to music^7^,^8^. However, we did not observe significant differences in the activity elicited by human or artificial voices in these auditory association areas, including the voice-sensitive area in STG^9^,^10^, suggesting that auditory association areas process human and artificial voices in the same manner.

Only the left posterior insular cortex revealed marked differences in the activity elicited by human or artificial voices, with higher activation elicited by the human voice. Although the posterior insula has reciprocal anatomical associations with the auditory cortex, temporal pole, and STG^11^,^12^, the functional role of this region in auditory processing remains unclear. The insula activation in the present study did not reflect a difference in the acoustic features between human and artificial voices. Moreover, the participants unable to detect a difference between human and artificial voices showed increased activation in the insular cortex in response to the human voice compared with the artificial voice. These results suggest that activity in the posterior insula reflects a sense of humanness with voice. Consistent with our findings, a previous electroencephalogram study demonstrated that neural responses to mismatches between human and artificial voices correspond to the holistic prototypical representation of human voice rather than to the stimulus acoustic features^13^. Furthermore, an electrophysiological study in monkeys revealed that the posterior insular cortex preferentially responds to conspecific vocalizations rather than vocalizations of other animals or environmental sounds^14^. Altogether, these findings indicate that the posterior insular cortex is involved in the processing of the ecological quality of a conspecific voice.

The present behavioral study showed that the human voice elicited a sense of humanness and positive feelings in the participants. Considering that the insula has dense associations with the amygdala^12^, the associations between the insula and amygdala may serve in associating vocal communication with emotions^11^. Therefore, the left posterior insula is believed to be involved not only in evaluating the ecological quality of human vocalization but also in eliciting a positive emotional response. However, the present study could not explain whether the sense of humanness in the voice induced positive feelings or vice versa. The causal relationship between the sense of humanness and positive feelings as well as the underlying neural mechanisms should be investigated further. Moreover, the sense of humanness is constructed from elements, such as visual appearance, voice, behavior, and touch. Future studies require to clarify whether the insula is involved in the sense of humanness not only with or voice but also with other modalities.

## Methods

### Auditory Stimuli

Participants were presented with songs sung by artificial voices that were created using the Vocaloid software (Yamaha Corporation, Hamamatsu, Japan). Vocaloid is a computer software with a library of actual human voice samples of all phonemes in a given language. These phonemes are assembled into a song phrase using the software's editing feature that allows the user to determine each phoneme note, duration, tone, and corresponding lyric, which the Vocaloid will sing. Further, users can modify the voice to sound more natural by adjusting parameters, such as dynamics, accents, vibrato, and breathiness. This software enables anyone to easily compose songs, and many amateur composers in Japan have used it for releasing original songs on the Internet. Such composers also provide the instrumental data of the song, allowing many amateur singers to cover it and post it online.

We selected 12 songs from the Internet for the experiments (6 pop songs and 6 ballads) sung by both artificial (Vocaloid software: GUMI sang 8 songs, IA sang 3 songs, and MIKU sang 1 song) and human voices (two singers sang 3 songs each, and six singers sang 1 song each). The instrumental accompaniment was identical between the artificial and human voice stimuli. All songs comprised Japanese lyrics sung in a female voice. We selected a 15-s segment containing lyrics from each song and created a total of 24 WAVE files for each experiment (sampling frequency: 44,100 Hz) with a tapering effect in the first and last seconds. The average (root mean square) power was adjusted such that there was no difference in the total power between human and artificial voices singing the same song. The music stimuli were presented at 70 ± 2.6 dB (background noise: 43 dB) from headphones. The study was approved by the institutional review board of Osaka University, and it was conducted in accordance with the approved guidelines. All participants provided written informed consent before participation.

### Spectrum Analysis

We first extracted the voice information from a song using a free available software (UTAGOE Lip, TODAKEN, Vector Inc., Tokyo, Japan). Next, the LTAS analysis was used to identify differences in acoustic features between human and artificial voices. LTAS was calculated from a narrow-band spectrogram (bandwidth: 40 Hz). LTAS was then normalized to the strongest peak and compared according to the differences in levels between the frequency ranges. The fundamental frequency was measured between 250 Hz and 700 Hz. The sound level for each frequency band was calculated with the equivalent sound level (Leq, dB). Further, the alpha ratio was calculated by subtracting the Leq in the 0.05–1-kHz range from that in the 1–5-kHz range [Leq (1−5 kHz/0.05−1 kHz) = Leq (1−5 kHz) – Leq (0.05–1 kHz)].

### Behavioral Experiment

#### Participants

Fourteen healthy participants (10 male, 4 female; median age: 28 years; range: 21–37 years) were included in the study. They had a normal hearing ability, and their experience in music or musical education was not considered. Before the experiment, each participant was asked what kind of music they listened to the most to determine whether they were familiar with artificial voices in music.

#### Procedure

The participants were in a sitting position, and auditory stimuli were presented through speakers (Computer MusicMonitor, Bose Corporation, Framingham, MA, USA) positioned in front of the participant. Each of the 24 song segments was presented once and in a random order, although the same song (sung by human and artificial voices) did not follow one another. The participants were not previously instructed that artificial singing voices were included in the stimuli.

After each song was presented once, the participants were sent in a quiet room where they immediately completed a questionnaire, wherein they rated the song from 1 to 5 on 10 different items in three categories. In order to determine whether the participants noticed the difference between human and artificial voices (impression of human-likeness), the questionnaire asked the participants to rate humanness (1 = robotic, 5 = human-like), animateness (1 = inanimate, 5 = animate), and naturalness (1 = awkward, 5 = natural). Furthermore, to determine the internal positive feelings elicited by the song (impression of positive feelings), the questionnaire asked the participant to rate familiarity (1 = unfamiliar, 5 = familiar), warmth (1 = cold, 5 = warm), emotion (1 = unemotional, 5 = emotional), and comfort (1 = irritating, 5 = comfortable). The questionnaire also required the participants to rate the perception of the song's character according to the following: complexity (1 = simple, 5 = complex), regularity (1 = random, 5 = regular), and brightness (1 = dark, 5 = bright). These 3 items were set as a contrast to the other items because these items should not differ in the same song between human and artificial voices. In addition to the rating task, the participants were asked to answer whether they had heard the song before.

#### Data Analysis

To examine whether the songs sung by human voices can be separated from those sung by artificial voices by the responses on the questionnaires, we conducted a cluster analysis using MDS. The distance between each pair of songs was calculated by summing the differences in the scores on each questionnaire. With this matrix, we applied MDS to plot each song in a two-dimensional plane. We further examined whether we were able to distinguish human and artificial voices in the MDS plane using a linear discriminant analysis. Next, we conducted an exploratory factor analysis with varimax rotation to investigate the factor underlying the questionnaire items.

### FMRI experiment

#### Participants

Eighteen healthy young adults (9 male, 9 female; median age: 21 years; range: 20–26 years) participated in this study. All had normal or corrected-to-normal vision with no history of auditory or neurological disorders. Before the experiment, the musical preference of each participant was enquired to ensure that they were not familiar with artificial voices in music.

#### Procedures

The experiment comprised two sessions. In each 390-s session, 12 songs were presented (6 human voices, 6 artificial voices) to the participants through headphones. The order of the songs was randomized. Visual stimuli were projected onto a screen outside the MRI magnet that the participants saw through a mirror. They were not informed that the stimuli contained songs sung by artificial voices.

Each session began with a 30-s pre-experimental block, followed by 12 cycles of a 15-s song block, 5-s response block, and 10-s rest block. After the final rest block, the participants completed a 15-s post-experimental block. While the song was presented, the screen presented instructions for the task (“Is this song human-like or robotic?”), and during the response block, the rating scale was presented to the participants to answer the questions. The stimuli presentation was controlled using the Presentation software (Neurobehavioral Systems, Inc., Berkeley, CA, USA).

After each song was played, the participants rated the humanness of the song on a scale of 1 to 5 (1: very robotic; 5: very human-like). We selected this question because the rating for the item “humanness” showed the greatest difference between human and artificial voices in our behavioral experiment. The participants were asked to hold a button in their right hand during the experiment and to answer the questions by pressing the button.

#### Data Acquisition

Structural images were collected for each participant using a T1-weighted, three-dimensional magnetization-prepared 180° radio-frequency pulse and rapid gradient-echo sequence on a Siemens 3-Tesla whole-body scanner with a 32-channel head coil [TR: 1.9 s; TE, 2.5 ms; flip angle: 9°; field of view: 256 mm; resolution: 1 × 1 × 1 mm). Functional images were collected using a gradient echo, echo-planar sequence (TR: 3 s; TE: 30 ms; flip angle: 80°; isotropic nominal resolution: 3 mm; 32 adjacent contiguous slices with no gap).

#### Data analysis

In the analysis, we excluded 5 participants (4 male, 1 female) because they could not distinguish between human and artificial voices under the fMRI background noise, and this was determined by the results of the rating task (paired *t*-test, p > 0.05). For the other 13 participants, the mean rating of humanness for the human voice was significantly higher than that for the artificial voice (paired *t*-test, p < 0.05). The mean ratings provided by the 13 participants revealed a significant difference between the human and artificial voices (human voice: 3.9 ± 0.3; artificial voice: 2.2 ± 0.3; p < 0.00001, paired *t*-test).

We used SPM8 (Wellcome Trust Centre for Neuroimaging) for data processing, which included realignment for head motion correction, normalization to the standard brain template (MNI template), smoothing with an 8-mm full-width half-maximum Gaussian filter, and statistical analyses. The statistical significance of brain activation was evaluated based on the voxel-wise signal changes with a general linear model with the standard hemodynamic function of SPM and random effects analysis. We set the threshold of significance to p < 0.001 (voxel level, uncorrected) and the extent of cluster size (k) >20 based on a previous study^15^. We used a one-sample *t*-test to identify the regions activated by human and artificial voices. To determine the difference in activation between human and artificial voices, we used a paired *t*-test.

Next, we analyzed the temporal dynamics of the BOLD signal changes in response to the auditory stimuli. We extracted the time courses of the signal intensities in each voxel in the region of interest. The time series was high-pass filtered (cut-off cycle: 128 s), converted to z-score, and linearly interpolated at a 1-s resolution. The time course was averaged separately for human and artificial voices in each participant and then averaged across participants.

## Author Contributions

T.N., Y.T. and S.K. wrote the main manuscript text and prepared the figures. All of the authors reviewed the manuscript.

## Supplementary Material

Supplementary Information Supplementary information

## Figures and Tables

**Table 1:** Difference in the average long-term average spectrum (LTAS) and alpha ratio between the human (n = 12) and artificial (n = 12) voices

		0–1 kHz	1–2 kHz	2–3 kHz	3–4 kHz	4–5 kHz	>5 kHz	Alpha ratio
Human voice								
Mean		−6.9 dB	−11.3 dB	−16.9 dB	−18.2 dB	−20.7 dB	−32.0 dB	−1.9 dB
SD		1.0	2.7	5.4	5.0	6.4	3.1	3.0
Artificial voice								
Mean		−7.9 dB	−13.2 dB	−22.5 dB	−21.3 dB	−25.9 dB	−34.7 dB	−3.8 dB
SD		0.9	3.4	5.5	5.1	4.1	4.7	3.4
Paired t-test		*p* = 0.02	*p* = 0.1	*p* = 0.02	*p* = 0.2	*p* = 0.03	*p* = 0.1	*p* = 0.2

SD, standard deviation.

**Table 2:** Mean impression score for the 10 items about the song stimuli (*p < 0.005 after Bonferroni correction)

	Factor		Human voice		Artificial voice		*t*-test		
	1	2	mean	SD	mean	SD	t value	p	significance
Humanness	**0.87**	0.23	4.0	0.4	2.5	0.5	7.6	p < 10^−9^	*
Animateness	**0.89**	0.23	3.8	0.4	2.6	0.5	6.5	p < 10^−9^	*
Naturalness	**0.91**	0.28	3.8	0.5	2.7	0.6	5.5	p = 0.0001	*
Emotional	**0.88**	0.32	3.8	0.3	2.8	0.5	6.7	p < 10^−9^	*
Familiarity	**0.82**	0.39	3.8	0.2	3.1	0.4	5.4	p < 10^−9^	*
Comfort	**0.76**	0.44	3.7	0.3	3.2	0.4	5.1	p = 0.0002	*
Warmth	**0.86**	−0.06	3.5	0.3	3.1	0.4	3.5	p < 0.004	*
Brightness	0.35	−0.14	3.3	0.4	3.0	0.3	2.5	p = 0.03	n.s.
Complexity	−0.02	**0.95**	2.9	0.4	2.8	0.5	1.0	p = 0.3	n.s.
Regularity	−0.29	−**0.73**	3.2	0.4	3.5	0.3	−2.1	p = 0.06	n.s.

SD, standard deviation; n.s., not significant.

**Table 3:** Brain regions showing activation in response to human (a) and artificial (b) voices as well as areas showing significantly greater activation for a human voice compared to an artificial voice (c) and vice versa (d)

		MNI coordinates				
Anatomical region	Laterality	x	y	z	t value	Z value
***(a) Human voice > Rest***						
Superior temporal gyrus	R	64	−18	8	18.1	6.2
	L	−48	0	−12	17.0	6.1
Inferior frontal gyrus	R	54	8	24	9.8	5.1
	L	−58	8	24	10.2	5.1
	L	−44	32	−2	8.0	4.6
Supplementary motor area	R	6	16	48	13.7	5.7
	L	−2	6	64	9.0	4.9
Cerebellum	R	28	−58	−28	12.4	5.5
	L	−30	−62	−24	8.0	4.6
Insular	R	34	20	4	6.7	4.2
	L	−26	18	0	11.8	5.4
Precentral gyrus	R	54	0	50	9.1	4.9
	L	−50	−6	50	11.4	5.4
Putamen	R	16	−2	−6	6.3	4.1
	L	−20	−2	10	8.3	4.7
***(b) Artificial voice > Rest***						
Superior temporal gyrus	R	54	−14	4	18.1	6.2
	L	−52	6	−10	16.1	6.0
Inferior frontal gyrus	R	54	8	22	10.2	5.1
	L	−44	16	8	9.7	5
Supplementary motor area	R	6	16	48	13.5	5.7
	L	−8	12	50	8.9	4.9
Cerebellum	R	28	−58	−28	7.7	4.5
	L	−30	−62	−26	7.1	4.4
Insula	L	−28	18	0	9.5	5.0
Precentral gyrus	R	52	0	50	12.2	5.5
	L	−48	−6	48	11.8	5.4
Putamen	R	26	20	−4	9	4.9
	L	−22	−6	12	8.1	4.7
***(c) Human voice > Artificial voice***						
Insula	L	−38	0	−6	6.4	4.1
***(d) Artificial voice > Human voice***						
no region						

MNI, Montreal Neurological Institute; R, right; L, left.
