# Supplementary material for: Involvement of the left insula in the ecological validity of the human voice
Source: Sci Rep. 2015 Mar 5;5:8799. doi: 10.1038/srep08799 (PMC4350090; doi:10.1038/srep08799)

Supplementary Information

Involvement of the left insula in fine discrimination of ecological quality of human voice

Yuri Tamura, Shinji Kuriki, Tamami Nakano*

Dynamic Brain Network Laboratory, Graduate School of Frontiers Biosciences, Osaka University, Osaka, Japan

*Correspondence to Tamami Nakano (tamami_nakano@fbs.osaka-u.ac.jp)

Supplementary Figure S1

Time course of the insula activation in response to the human voice against the artificial voices. The magenta line represents the time course average across participants included in the fMRI analysis (n=13) and the green line represents the time course averaged across participants excluded from the fMRI analysis (n=5).

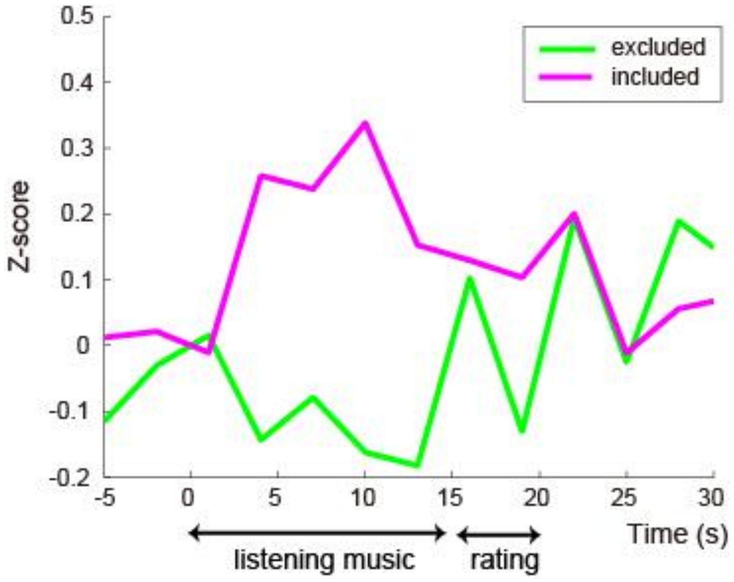

Supplement: Supplementary Information [file srep08799-s1.pdf]
